# Editorial: Pattern Recognition Receptors and Cancer

**DOI:** 10.3389/fimmu.2015.00481

**Published:** 2015-09-16

**Authors:** Anton G. Kutikhin, Arseniy E. Yuzhalin

**Affiliations:** ^1^Laboratory for Genomic Medicine, Division of Experimental and Clinical Cardiology, Research Institute for Complex Issues of Cardiovascular Diseases under the Siberian Branch of the Russian Academy of Medical Sciences, Kemerovo, Russia; ^2^Cancer Research UK/MRC Oxford Institute for Radiation Oncology, Department of Oncology, University of Oxford, Oxford, UK

**Keywords:** toll-like receptors, NOD-like receptors, C-type lectin receptors, RIG-I-like receptors, pattern recognition receptors, innate immunity, inflammation, cancer

The problem of cancer remains one of the most immense challenges to current biomedical research. Affecting populations in all countries and all regions, this disease is responsible for millions of deaths annually (1). Evasion of the immune system is an ominous feature of cancers, which often leads to tumor outgrowth, epithelial–mesenchymal transition (EMT), and consequently, metastatic disease. The need to understand basic mechanisms governing immune response to tumors is increasingly acute, since contemporary cancer research gradually progresses toward highly specialized personalized medicine. In this respect, oncoimmunology of pattern recognition receptors (PRRs) is a promising area of research which requires more attention and broader interpretation.

The group of PRRs includes families of toll-like receptors (TLRs), NOD-like receptors (NLRs), C-type lectin receptors (CLRs), RIG-I-like receptors (RLRs), and AIM-2-like receptors (ALRs). United by two general features, these receptors are the key players in human immunity. First, they directly recognize antigen determinants of nearly all classes of pathogens [pathogen-associated molecular patterns (PAMPs)] and promote their elimination by triggering innate and adaptive immune response. Second, they recognize endogenous ligands released during cell stress [damage-associated molecular patterns (DAMPs)], and therefore can activate immune response in the absence of an infectious agent. In addition, PRRs are known to possess a number of other vital functions, regulating the processes of apoptosis, DNA repair, autophagy, and angiogenesis. Remarkable functional significance and diversity of biological functions are the reasons why PRRs today are an actively growing area of research.

During the last decade, much research has been done to investigate the role of PRRs in tumor immunity. Accumulating evidence demonstrate that anti-tumor immunity can be stimulated through the activation of PRRs (2, 3). It has been repeatedly shown that reinforced PRR activation may protect the host from infectious agents and prevent, inhibit, or block carcinogenesis whereas disrupted or deregulated functioning of PRRs may promote cancer through weakening the immune system (2, 3). At the same time, PRR activation may stimulate cancer by creating a proinflammatory microenvironment which is favorable for tumor progression and chemoresistance development (4). Furthermore, it may also result in immunosuppression caused by chronic inflammation (2), which is known to promote the development of breast carcinoma, colorectal cancer, pancreatic adenocarcinoma, and possibly several other cancer types (5, 6). In this case, on the contrary, lower PRR activity should minimize effects of chronic inflammation such as enhancement of cancer initiation and promotion/progression and, consequently, decrease probability of tumor development (4). Therefore, the situation resembles a double-edged sword, where both sides can cut unless golden mean is maintained. In this respect, it is clear that a subtle balance of low and high PRR activity is required for proper functioning of the immune system. This hypothesis, initially developed for PRRs (3), may also be successfully projected on PRR intracellular signaling pathways – if their elements are overexpressed/constantly activated, it may lead to consequences similar to that of enhanced PRR activation (7, 8). On the other hand, if downstream members of PRR pathways are underexpressed, inactivated, or unable to work properly, it may result in the same effects that of diminished PRR activity, and therefore a balance in functioning of all genes encoding proteins constituting PRR signaling pathways should be preserved for optimal immune system function (7, 8).

Three years ago, four milestone reviews on PRR biology were published in *Immunity* (9–12); we now think that *Frontiers in Immunology* can be an excellent platform for the constellation of review articles systematizing key information in the field with regard to the recent discoveries. With this aim in mind, we invited a number of recognized experts in the field to submit review papers on various aspects of PRR biology and their role in cancer. We sincerely thank all researchers who have agreed to contribute to our Research Topic.

This collection is divided into three sections. The first section describes basic functions of PRRs along with their signaling pathways, and was established with the participation of Taro Kawai and colleagues, Mansi Saxena and Garabet Yeretssian, Huimin Yan and colleagues, together with Stephanie Reikine, Jennifer Nguyen, and Yorgo Modis. We also sought to solicit a number of additional review articles on TLR and NLR biology, since we believe these topics deserve a particular attention. Regarding TLRs, Ajay Jain, Sabina Kaczanowska, and Eduardo Davila depict the newest schemes of the IL-1 receptor-associated kinase signaling, whereas Asif Amin Dar, Rushikesh Sudam Patil, and Shubhada Vivek Chiplunkar provide the insights into the relationship between TLRs and γδ T cell response. Readers interested in NLR structure and functioning will definitely appreciate elegant papers by Irving Coy Allen, Julie Magarian Blander, and Andrew Kent together with Silvia Lucena Lage and colleagues. In addition, a brilliant review by Nelson Di Paolo fills a substantial gap in the understanding of the recognition of human oncogenic viruses by PRRs. Finally, Raunaq Singh Nagi, Ashish Bhat, and Himanshu Kumar close up the first section with the description of the general conception on the role of PRRs in cancer development.

The second section is devoted to the role of PRRs in various vital cellular processes, including apoptosis, DNA repair, autophagy, and angiogenesis. It is contributed by Gustavo Amarante-Mendes and colleagues, Anton Kutikhin and colleagues, Ji Eun Oh, and Heung Kyu Lee along with Sheeba Murad.

Finally, the last piece of the collection consists of reviews that comprehensively analyze the impact of PRRs on the development of malignant tumors (esophageal cancer, gastric cancer, colorectal cancer, lung cancer, prostate cancer, breast cancer, ovarian cancer, and lymphoma). Furthermore, Simon Heidegger and colleagues discuss the role of PRRs in graft-versus-host disease and graft-versus-leukemia following allogeneic stem cell transplantation. As a final point, Shanjana Awasthi underlines the importance of TLR agonists in cancer immunotherapy.

We created this Research Topic with the hope that it will be useful for a wide audience, particularly cancer researchers, immunologists, microbiologists, graduate, and undergraduate students of biomedical faculties as well as for their lecturers.

## Conflict of Interest Statement

The authors declare that the research was conducted in the absence of any commercial or financial relationships that could be construed as a potential conflict of interest.
